# *cis*-(Acetonitrile-κ*N*)aqua­bis­(2,2′-bipyrimidine-κ^2^ *N* ^1^,*N* ^1′^)manganese(II) *cis*-diaqua­bis­(2,2′-bipyrimidine-κ^2^ *N* ^1^,*N* ^1′^)manganese(II) tetrakis(perchlorate) dihydrate

**DOI:** 10.1107/S1600536811015388

**Published:** 2011-05-07

**Authors:** Kwang Ha

**Affiliations:** a School of Applied Chemical Engineering, The Research Institute of Catalysis, Chonnam National University, Gwangju 500-757, Republic of Korea

## Abstract

The asymmetric unit of the title compound, [Mn(CH_3_CN)(C_8_H_6_N_4_)_2_(H_2_O)][Mn(C_8_H_6_N_4_)_2_(H_2_O)_2_](ClO_4_)_4_·2H_2_O, contains two distinct cationic Mn^II^ complexes, four perchlorate anions and two solvent water mol­ecules. In the two complexes, both Mn^II^ ions are six-coordinated in a distorted octa­hedral environment, but one Mn ion has a *cis*-N_5_O coordination geometry defined by four N atoms of the two chelating 2,2′-bipyrimidine (bpym) ligands, one N atom of a coordinating acetonitrile mol­ecule and one O atom of a water ligand, whereas the other Mn ion has a *cis*-N_4_O_2_ coordination geometry defined by four N atoms of the two bpym ligands and two O atoms of water ligands. In the crystal structure, the complex mol­ecules, anions and solvent water mol­ecules are linked by inter­molecular O—H⋯O and O—H⋯N hydrogen bonds. Three of the four perchlorate anions are disordered over two sets of sites in different ratios.

## Related literature

For the crystal structures of mononuclear 2,2′-bipyrimidine Mn(II) complexes, see: Hong *et al.* (1996 ▶); Smith *et al.* (2001 ▶); Ha (2011 ▶).
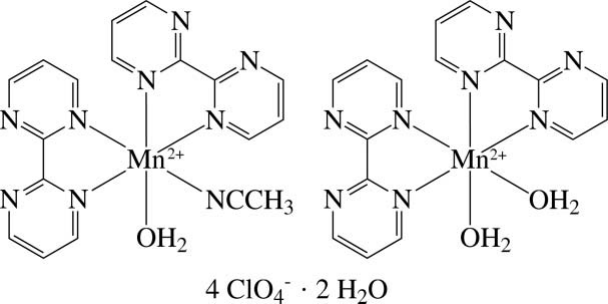


## Experimental

### 

#### Crystal data

[Mn(C_2_H_3_N)(C_8_H_6_N_4_)_2_(H_2_O)][Mn(C_8_H_6_N_4_)_2_(H_2_O)_2_](ClO_4_)_4_·2H_2_O
*M* *_r_* = 1271.49
Triclinic, 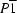

*a* = 12.0386 (5) Å
*b* = 13.1878 (6) Å
*c* = 17.5378 (8) Å
α = 111.201 (3)°
β = 104.147 (3)°
γ = 91.419 (2)°
*V* = 2497.37 (19) Å^3^
*Z* = 2
Mo *K*α radiation
μ = 0.82 mm^−1^
*T* = 200 K
0.24 × 0.21 × 0.13 mm

#### Data collection

Bruker SMART 1000 CCD diffractometer
Absorption correction: multi-scan (*SADABS*; Bruker, 2000 ▶) *T*_min_ = 0.765, *T*_max_ = 0.899
18759 measured reflections
12197 independent reflections
5402 reflections with *I* > 2σ(*I*)
*R*_int_ = 0.052

#### Refinement

*R*[*F* ^2^ > 2σ(*F* ^2^)] = 0.082
*wR*(*F* ^2^) = 0.253
*S* = 1.01
12197 reflections
718 parameters
H-atom parameters constrained
Δρ_max_ = 1.01 e Å^−3^
Δρ_min_ = −0.61 e Å^−3^

### 

Data collection: *SMART* (Bruker, 2000 ▶); cell refinement: *SAINT* (Bruker, 2000 ▶); data reduction: *SAINT*; program(s) used to solve structure: *SHELXS97* (Sheldrick, 2008 ▶); program(s) used to refine structure: *SHELXL97* (Sheldrick, 2008 ▶); molecular graphics: *ORTEP-3* (Farrugia, 1997 ▶) and *PLATON* (Spek, 2009 ▶); software used to prepare material for publication: *SHELXL97*.

## Supplementary Material

Crystal structure: contains datablocks global, I. DOI: 10.1107/S1600536811015388/om2423sup1.cif

Structure factors: contains datablocks I. DOI: 10.1107/S1600536811015388/om2423Isup2.hkl

Additional supplementary materials:  crystallographic information; 3D view; checkCIF report

## Figures and Tables

**Table:** 

Mn1—N9	2.170 (6)
Mn1—O1	2.182 (4)
Mn1—N4	2.236 (4)
Mn1—N8	2.254 (5)
Mn1—N1	2.259 (5)
Mn1—N5	2.271 (5)
Mn2—O3	2.107 (4)
Mn2—O2	2.174 (4)
Mn2—N13	2.238 (5)
Mn2—N10	2.267 (5)
Mn2—N14	2.269 (5)
Mn2—N17	2.272 (5)

**Table:** 

N4—Mn1—N1	73.12 (18)
N8—Mn1—N5	73.27 (18)
N13—Mn2—N10	72.99 (19)
N14—Mn2—N17	72.26 (18)

**Table 2:** Hydrogen-bond geometry (Å, °)

*D*—H⋯*A*	*D*—H	H⋯*A*	*D*⋯*A*	*D*—H⋯*A*
O1—H1*A*⋯N6^i^	0.84	2.49	3.139 (6)	135
O1—H1*A*⋯N7^i^	0.84	2.15	2.923 (6)	152
O1—H1*B*⋯O21^i^	0.84	1.89	2.695 (7)	160
O2—H2*A*⋯N15^ii^	0.84	2.59	3.244 (7)	136
O2—H2*A*⋯N16^ii^	0.84	2.25	3.012 (7)	151
O2—H2*B*⋯O11*A*^iii^	0.84	2.01	2.809 (10)	158
O3—H3*A*⋯O20	0.84	1.79	2.614 (6)	166
O3—H3*B*⋯O4^iii^	0.84	2.07	2.861 (6)	157
O20—H20*A*⋯N2^iv^	0.84	2.08	2.903 (7)	168
O20—H20*A*⋯N3^iv^	0.84	2.56	3.066 (7)	120
O20—H20*B*⋯O6	0.84	2.08	2.871 (7)	157
O21—H21*A*⋯O12^v^	0.84	2.43	3.126 (12)	141
O21—H21*B*⋯O16*A*^iv^	0.84	2.07	2.904 (13)	172

Symmetry codes: (i) 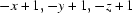; (ii) 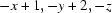; (iii) 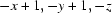; (iv) 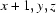; (v) 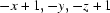.

**Fig. 1.**
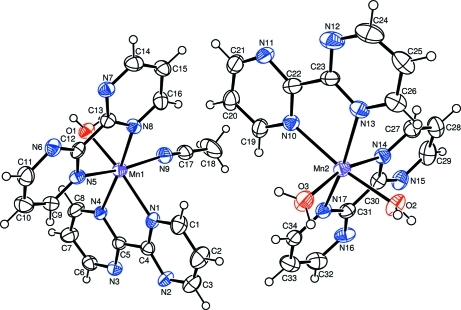
The structure of the title compound, with displacement ellipsoids drawn at the 40% probability level for non-H atoms. The four ClO4- anions and the two H2O solvent molecules have been omitted for clarity.

**Fig. 2.**
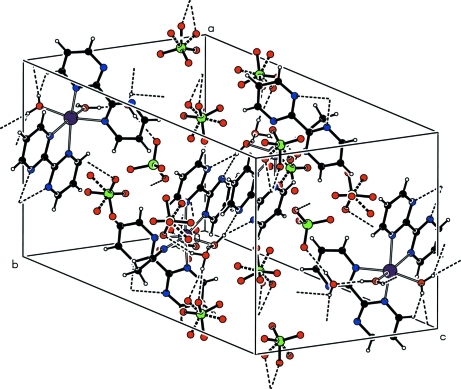
View of the unit-cell contents of the title compound. Hydrogen-bond interactions and the bonds of the disordered anions are drawn with dashed lines.

## supplementary crystallographic information

### Comment

Mononuclear Mn^II^ complexes of 2,2'-bipyrimidine (bpym; C_8_H_6_N_4_) ligand, such as [Mn(bpym)_2_(H_2_O)_2_](ClO_4_)_2_.2H_2_O (Hong *et al.*, 1996), [Mn(bpym)_2_(H_2_O)_2_](BF_4_)_2_.2H_2_O (Smith *et al.*, 2001) and [MnBr_2_(bpym)_2_].CH_3_NO_2_ (Ha, 2011), have been investigated previously.

The asymmetric unit of the title compound, [Mn(bpym)_2_(CH_3_CN)(H_2_O)][Mn(bpym)_2_(H_2_O)_2_](ClO_4_)_4_.2H_2_O, contains two distinct cationic Mn^II^ complexes (Fig. 1), four ClO_4_ anions and two solvent water molecules. In the two complexes, both the Mn^II^ ions are six-coordinated in a distorted octahedral environment, but one Mn ion has a *cis*-N_5_O coordination geometry defined by four N atoms of the two chelating 2,2'-bipyrimidine ligands, one N atom of a coordinating acetonitrile molecule and one O atom of a water ligand, whereas the other Mn ion has a *cis*-N_4_O_2_ coordination geometry defined by four N atoms of the two bpym ligands and two O atoms of water ligands. The tight N—Mn—N chelating angles (Table 1) contribute the distortion of the octaahedron, which results in non-linear *trans* axes (<O1—Mn1—N1 = 163.1 (2)°, <N4—Mn1—N8 = 172.3 (2)°, <N5—Mn1—N9 = 165.5 (2)°, <O2—Mn2—N10 = 165.9 (2)°, <O3—Mn2—N14 = 162.0 (2)° and <N13—Mn2—N17 = 161.2 (2)°). The Mn—N(bpym) bond lengths are almost equivalent and slightly longer than the Mn1—N9(CH_3_CN) bond (Table 1). On the basis of the Mn—N bonds *trans* to the N and O atoms, it seems that the N and O atoms have similar *trans* effects. The dihedral angles between the least-squares planes of the two bpym ligands in the respective complexes are 79.7 (1)° and 70.7 (1)°. In the crystal structure, the complexes, anions and solvent water molecules are linked by intermolecular O—H···O and O—H···N hydrogen bonds (Fig. 2, Table 2). In addition, the complexes display numerous inter- and intramolecular π-π interactions between adjacent pyrimidine rings. The shortest distance between *Cg*1 (the centroid of ring N14—C30) and *Cg*2^i^ (ring N16—C34, symmetry code i: 1 - *x*, 2 - *y*, -*z*) is 3.585 (4) Å, and the dihedral angle between the ring planes is 3.3 (3)°.

### Experimental

To a solution of Mn(ClO_4_)_2_.6H_2_O (0.3617 g, 0.999 mmol) in EtOH (20 ml) was added 2,2'-bipyrimidine (0.1586 g, 1.003 mmol) and stirred for 3 h at room temperature. The formed precipitate was separated by filtration, washed with EtOH and dried at 323 K, to give a pale yellow powder (0.2713 g). Crystals suitable for X-ray analysis were obtained by slow evaporation from a CH_3_CN solution.

### Refinement

H atoms were positioned geometrically and allowed to ride on their respective parent atoms [C—H = 0.95 Å (CH) or 0.98 Å (CH_3_), O—H = 0.84 Å and *U*_iso_(H) = 1.2*U*_eq_(C) or 1.5*U*_eq_(methyl C, O)]. The ClO_4_^-^ anions displayed relatively large displacement factors and low electron density peaks so that the anions appear to be highly disordered. Atoms O10 and O11 were modelled anisotropically as disordered over two sites with a site occupancy factor of 0.71 (3) for the major component. However, the seven O atoms (O13–O19) of the two ClO_4_^-^ anions were refined with isotropic thermal parameters as disordered over two sites with site occupancy factors of 0.61 (1) for the atoms O13A–O15A and 0.54 (2) for the atoms O16A–O19A. The large *R* values are connected with the disorder of the anions and the isotropic refinement of the O atoms.

### Crystal data

**Table:**

[Mn(C_2_H_3_N)(C_8_H_6_N_4_)_2_(H_2_O)][Mn(C_8_H_6_N_4_)_2_(H_2_O)_2_](ClO_4_)_4_·2H_2_O	*Z* = 2
*M**_r_* = 1271.49	*F*(000) = 1292
Triclinic, *P*1	*D*_x_ = 1.691 Mg m^−^^3^
Hall symbol: -P 1	Mo *K*α radiation, λ = 0.71073 Å
*a* = 12.0386 (5) Å	Cell parameters from 3208 reflections
*b* = 13.1878 (6) Å	θ = 2.3–23.0°
*c* = 17.5378 (8) Å	µ = 0.82 mm^−^^1^
α = 111.201 (3)°	*T* = 200 K
β = 104.147 (3)°	Block, pale yellow
γ = 91.419 (2)°	0.24 × 0.21 × 0.13 mm
*V* = 2497.37 (19) Å^3^	

### Data collection

**Table:**

Bruker SMART 1000 CCD diffractometer	12197 independent reflections
Radiation source: fine-focus sealed tube	5402 reflections with *I* > 2σ(*I*)
graphite	*R*_int_ = 0.052
φ and ω scans	θ_max_ = 28.3°, θ_min_ = 1.8°
Absorption correction: multi-scan (*SADABS*; Bruker, 2000)	*h* = −15→16
*T*_min_ = 0.765, *T*_max_ = 0.899	*k* = −17→17
18759 measured reflections	*l* = −13→23

### Refinement

**Table:**

Refinement on *F*^2^	Primary atom site location: structure-invariant direct methods
Least-squares matrix: full	Secondary atom site location: difference Fourier map
*R*[*F*^2^ > 2σ(*F*^2^)] = 0.082	Hydrogen site location: inferred from neighbouring sites
*wR*(*F*^2^) = 0.253	H-atom parameters constrained
*S* = 1.01	*w* = 1/[σ^2^(*F*_o_^2^) + (0.1047*P*)^2^] where *P* = (*F*_o_^2^ + 2*F*_c_^2^)/3
12197 reflections	(Δ/σ)_max_ < 0.001
718 parameters	Δρ_max_ = 1.01 e Å^−^^3^
0 restraints	Δρ_min_ = −0.61 e Å^−^^3^

### Special details

**Table:**

Geometry. All e.s.d.'s (except the e.s.d. in the dihedral angle between two l.s. planes) are estimated using the full covariance matrix. The cell e.s.d.'s are taken into account individually in the estimation of e.s.d.'s in distances, angles and torsion angles; correlations between e.s.d.'s in cell parameters are only used when they are defined by crystal symmetry. An approximate (isotropic) treatment of cell e.s.d.'s is used for estimating e.s.d.'s involving l.s. planes.
Refinement. Refinement of *F*^2^ against ALL reflections. The weighted *R*-factor *wR* and goodness of fit *S* are based on *F*^2^, conventional *R*-factors *R* are based on *F*, with *F* set to zero for negative *F*^2^. The threshold expression of *F*^2^ > σ(*F*^2^) is used only for calculating *R*-factors(gt) *etc*. and is not relevant to the choice of reflections for refinement. *R*-factors based on *F*^2^ are statistically about twice as large as those based on *F*, and *R*- factors based on ALL data will be even larger.

### Fractional atomic coordinates and isotropic or equivalent isotropic displacement parameters (Å^2^)

**Table:**

	*x*	*y*	*z*	*U*_iso_*/*U*_eq_	Occ. (<1)
Mn1	0.24790 (7)	0.60902 (8)	0.39169 (6)	0.0372 (3)	
O1	0.2664 (3)	0.6133 (3)	0.5198 (3)	0.0432 (11)	
H1A	0.3205	0.5802	0.5356	0.065*	
H1B	0.2764	0.6812	0.5477	0.065*	
N1	0.1739 (4)	0.6075 (4)	0.2598 (3)	0.0364 (12)	
N2	−0.0030 (4)	0.5887 (4)	0.1555 (3)	0.0441 (13)	
N3	−0.1202 (4)	0.5380 (4)	0.2547 (3)	0.0396 (12)	
N4	0.0595 (4)	0.5495 (4)	0.3538 (3)	0.0362 (12)	
N5	0.3165 (4)	0.4446 (4)	0.3495 (3)	0.0368 (12)	
N6	0.4890 (4)	0.3620 (4)	0.3555 (3)	0.0429 (13)	
N7	0.6126 (4)	0.5630 (4)	0.4485 (3)	0.0393 (12)	
N8	0.4414 (4)	0.6449 (4)	0.4233 (3)	0.0355 (12)	
N9	0.2262 (5)	0.7817 (5)	0.4347 (4)	0.0513 (15)	
C1	0.2310 (6)	0.6318 (6)	0.2120 (5)	0.0525 (18)	
H1	0.3127	0.6474	0.2315	0.063*	
C2	0.1743 (7)	0.6353 (7)	0.1334 (5)	0.068 (2)	
H2	0.2159	0.6518	0.0989	0.081*	
C3	0.0567 (6)	0.6138 (6)	0.1085 (5)	0.058 (2)	
H3	0.0159	0.6168	0.0558	0.069*	
C4	0.0589 (5)	0.5865 (5)	0.2290 (4)	0.0358 (14)	
C5	−0.0048 (5)	0.5562 (5)	0.2814 (4)	0.0333 (13)	
C6	−0.1735 (5)	0.5090 (5)	0.3031 (4)	0.0443 (16)	
H6	−0.2555	0.4963	0.2862	0.053*	
C7	−0.1166 (5)	0.4966 (6)	0.3754 (4)	0.0546 (19)	
H7	−0.1560	0.4737	0.4083	0.065*	
C8	0.0035 (5)	0.5196 (6)	0.3986 (5)	0.0553 (19)	
H8	0.0465	0.5132	0.4494	0.066*	
C9	0.2540 (5)	0.3463 (5)	0.3085 (4)	0.0438 (16)	
H9	0.1721	0.3410	0.2927	0.053*	
C10	0.3062 (6)	0.2534 (6)	0.2891 (4)	0.0526 (18)	
H10	0.2623	0.1830	0.2597	0.063*	
C11	0.4237 (6)	0.2647 (6)	0.3134 (4)	0.0509 (18)	
H11	0.4611	0.2005	0.2997	0.061*	
C12	0.4308 (5)	0.4484 (5)	0.3712 (4)	0.0351 (14)	
C13	0.4997 (5)	0.5586 (5)	0.4168 (4)	0.0343 (14)	
C14	0.6714 (5)	0.6624 (6)	0.4895 (4)	0.0486 (17)	
H14	0.7519	0.6686	0.5148	0.058*	
C15	0.6210 (5)	0.7575 (6)	0.4973 (4)	0.0464 (17)	
H15	0.6653	0.8278	0.5243	0.056*	
C16	0.5022 (6)	0.7446 (5)	0.4635 (4)	0.0483 (17)	
H16	0.4634	0.8076	0.4690	0.058*	
C17	0.1982 (5)	0.8652 (6)	0.4523 (5)	0.0510 (18)	
C18	0.1624 (7)	0.9751 (6)	0.4753 (6)	0.092 (3)	
H18A	0.2310	1.0300	0.5008	0.138*	
H18B	0.1169	0.9855	0.5163	0.138*	
H18C	0.1155	0.9835	0.4243	0.138*	
Mn2	0.73069 (7)	0.84930 (7)	0.08687 (6)	0.0360 (3)	
O2	0.7368 (4)	0.8748 (4)	−0.0279 (3)	0.0487 (12)	
H2A	0.6912	0.9070	−0.0530	0.073*	
H2B	0.7646	0.8323	−0.0652	0.073*	
O3	0.7245 (4)	0.6807 (3)	0.0161 (3)	0.0546 (13)	
H3A	0.7333	0.6397	0.0438	0.082*	
H3B	0.7067	0.6516	−0.0372	0.082*	
N10	0.7711 (4)	0.8189 (4)	0.2094 (3)	0.0375 (12)	
N11	0.9225 (5)	0.8194 (4)	0.3254 (4)	0.0501 (15)	
N12	1.0769 (5)	0.8875 (5)	0.2568 (4)	0.0594 (17)	
N13	0.9206 (4)	0.8974 (4)	0.1473 (3)	0.0408 (13)	
N14	0.6835 (4)	1.0226 (4)	0.1326 (3)	0.0377 (12)	
N15	0.5259 (5)	1.1271 (5)	0.1331 (4)	0.0480 (14)	
N16	0.3769 (4)	0.9390 (5)	0.0614 (3)	0.0473 (14)	
N17	0.5367 (4)	0.8376 (4)	0.0686 (3)	0.0375 (12)	
C19	0.6952 (6)	0.7861 (5)	0.2431 (4)	0.0462 (16)	
H19	0.6153	0.7761	0.2149	0.055*	
C20	0.7279 (7)	0.7664 (5)	0.3158 (4)	0.0513 (18)	
H20	0.6738	0.7411	0.3384	0.062*	
C21	0.8441 (7)	0.7857 (5)	0.3543 (4)	0.0541 (19)	
H21	0.8699	0.7737	0.4057	0.065*	
C22	0.8816 (5)	0.8349 (5)	0.2529 (4)	0.0402 (15)	
C23	0.9659 (5)	0.8756 (5)	0.2170 (4)	0.0399 (15)	
C24	1.1476 (6)	0.9293 (7)	0.2243 (6)	0.070 (2)	
H24	1.2281	0.9400	0.2510	0.084*	
C25	1.1122 (6)	0.9575 (6)	0.1560 (5)	0.056 (2)	
H25	1.1650	0.9892	0.1360	0.067*	
C26	0.9945 (5)	0.9374 (5)	0.1170 (4)	0.0474 (17)	
H26	0.9660	0.9526	0.0673	0.057*	
C27	0.7571 (6)	1.1148 (5)	0.1646 (4)	0.0459 (16)	
H27	0.8378	1.1104	0.1753	0.055*	
C28	0.7185 (6)	1.2159 (6)	0.1824 (5)	0.0538 (18)	
H28	0.7708	1.2814	0.2046	0.065*	
C29	0.6023 (7)	1.2183 (6)	0.1669 (5)	0.061 (2)	
H29	0.5740	1.2875	0.1806	0.074*	
C30	0.5709 (5)	1.0339 (5)	0.1185 (4)	0.0386 (14)	
C31	0.4891 (5)	0.9305 (5)	0.0806 (4)	0.0378 (14)	
C32	0.3065 (6)	0.8437 (6)	0.0277 (4)	0.0546 (19)	
H32	0.2256	0.8457	0.0117	0.066*	
C33	0.3460 (6)	0.7444 (6)	0.0153 (4)	0.0535 (19)	
H33	0.2942	0.6788	−0.0060	0.064*	
C34	0.4637 (5)	0.7432 (5)	0.0351 (4)	0.0408 (15)	
H34	0.4941	0.6752	0.0250	0.049*	
Cl1	0.48977 (13)	0.50530 (13)	0.17253 (10)	0.0410 (4)	
O4	0.4008 (4)	0.4218 (4)	0.1592 (3)	0.0647 (14)	
O5	0.4494 (4)	0.5679 (5)	0.1233 (4)	0.0745 (16)	
O6	0.5876 (4)	0.4560 (4)	0.1486 (3)	0.0668 (15)	
O7	0.5234 (5)	0.5730 (5)	0.2605 (3)	0.0773 (17)	
Cl2	0.01877 (14)	0.26540 (14)	0.10338 (10)	0.0466 (4)	
O8	−0.0134 (4)	0.3198 (5)	0.1779 (3)	0.0739 (16)	
O9	−0.0433 (5)	0.2906 (6)	0.0345 (4)	0.093 (2)	
O10A	−0.0218 (13)	0.1481 (6)	0.0770 (5)	0.068 (4)	0.71 (3)
O11A	0.1384 (7)	0.2815 (11)	0.1164 (6)	0.068 (5)	0.71 (3)
O10B	0.077 (4)	0.178 (2)	0.0996 (19)	0.090 (18)	0.29 (3)
O11B	0.118 (4)	0.356 (4)	0.130 (3)	0.144 (16)	0.29 (3)
Cl3	0.52009 (15)	0.04352 (15)	0.66585 (13)	0.0599 (5)	
O12	0.4138 (6)	0.0080 (8)	0.6096 (6)	0.166 (4)	
O13A	0.5402 (8)	0.1556 (9)	0.6816 (7)	0.088 (4)*	0.613 (14)
O14A	0.6054 (10)	0.0031 (10)	0.6173 (8)	0.104 (4)*	0.613 (14)
O15A	0.5443 (13)	0.0240 (11)	0.7396 (9)	0.121 (5)*	0.613 (14)
O13B	0.5640 (15)	0.1420 (16)	0.7412 (14)	0.104 (7)*	0.387 (14)
O14B	0.5893 (11)	−0.0398 (11)	0.6507 (9)	0.061 (5)*	0.387 (14)
O15B	0.447 (2)	0.0083 (19)	0.7173 (16)	0.145 (10)*	0.387 (14)
Cl4	0.00909 (15)	0.18921 (14)	0.38159 (12)	0.0522 (5)	
O16A	−0.0859 (10)	0.1076 (10)	0.3297 (9)	0.081 (4)*	0.544 (15)
O17A	0.1037 (12)	0.1561 (12)	0.3431 (11)	0.119 (6)*	0.544 (15)
O18A	−0.0302 (12)	0.2824 (13)	0.3760 (10)	0.122 (6)*	0.544 (15)
O19A	0.0411 (15)	0.1971 (17)	0.4642 (12)	0.162 (8)*	0.544 (15)
O16B	−0.0760 (10)	0.0984 (10)	0.3678 (9)	0.060 (4)*	0.456 (15)
O17B	0.1208 (11)	0.1573 (11)	0.4020 (10)	0.080 (5)*	0.456 (15)
O18B	0.0048 (16)	0.2185 (17)	0.3119 (14)	0.146 (9)*	0.456 (15)
O19B	0.0154 (13)	0.2819 (15)	0.4580 (11)	0.106 (6)*	0.456 (15)
O20	0.7508 (4)	0.5264 (4)	0.0772 (3)	0.0555 (13)	
H20A	0.8204	0.5415	0.1055	0.083*	
H20B	0.7080	0.5248	0.1082	0.083*	
O21	0.6981 (4)	0.1840 (4)	0.3583 (3)	0.0710 (15)	
H21A	0.6973	0.1473	0.3886	0.106*	
H21B	0.7619	0.1613	0.3547	0.106*	

### Atomic displacement parameters (Å^2^)

**Table:**

	*U*^11^	*U*^22^	*U*^33^	*U*^12^	*U*^13^	*U*^23^
Mn1	0.0292 (5)	0.0412 (6)	0.0418 (6)	0.0069 (4)	0.0078 (4)	0.0175 (5)
O1	0.036 (2)	0.055 (3)	0.045 (3)	0.013 (2)	0.010 (2)	0.027 (2)
N1	0.038 (3)	0.041 (3)	0.039 (3)	0.011 (2)	0.018 (2)	0.021 (2)
N2	0.048 (3)	0.056 (4)	0.033 (3)	0.009 (3)	0.009 (3)	0.025 (3)
N3	0.034 (3)	0.042 (3)	0.042 (3)	0.006 (2)	0.011 (2)	0.015 (3)
N4	0.029 (3)	0.047 (3)	0.039 (3)	−0.003 (2)	0.007 (2)	0.025 (3)
N5	0.037 (3)	0.042 (3)	0.031 (3)	0.009 (2)	0.010 (2)	0.013 (2)
N6	0.042 (3)	0.043 (3)	0.049 (3)	0.012 (2)	0.013 (3)	0.022 (3)
N7	0.034 (3)	0.047 (3)	0.038 (3)	0.006 (2)	0.010 (2)	0.017 (3)
N8	0.031 (3)	0.040 (3)	0.037 (3)	0.000 (2)	0.013 (2)	0.014 (2)
N9	0.055 (4)	0.038 (3)	0.054 (4)	0.009 (3)	0.007 (3)	0.014 (3)
C1	0.048 (4)	0.060 (5)	0.061 (5)	0.011 (3)	0.023 (4)	0.032 (4)
C2	0.073 (5)	0.095 (6)	0.058 (5)	0.018 (5)	0.036 (5)	0.043 (5)
C3	0.069 (5)	0.071 (5)	0.041 (4)	0.015 (4)	0.017 (4)	0.028 (4)
C4	0.035 (3)	0.033 (3)	0.038 (4)	0.005 (3)	0.010 (3)	0.012 (3)
C5	0.034 (3)	0.031 (3)	0.031 (3)	0.005 (2)	0.007 (3)	0.009 (3)
C6	0.032 (3)	0.045 (4)	0.054 (4)	0.002 (3)	0.008 (3)	0.018 (3)
C7	0.039 (4)	0.079 (5)	0.049 (4)	−0.005 (4)	0.009 (3)	0.030 (4)
C8	0.039 (4)	0.079 (5)	0.054 (5)	−0.003 (3)	−0.001 (3)	0.042 (4)
C9	0.045 (4)	0.042 (4)	0.036 (4)	−0.002 (3)	0.002 (3)	0.013 (3)
C10	0.051 (4)	0.047 (4)	0.049 (4)	0.005 (3)	0.008 (4)	0.009 (3)
C11	0.065 (5)	0.039 (4)	0.051 (4)	0.015 (3)	0.020 (4)	0.016 (3)
C12	0.035 (3)	0.047 (4)	0.036 (3)	0.012 (3)	0.016 (3)	0.025 (3)
C13	0.025 (3)	0.042 (4)	0.041 (3)	0.008 (3)	0.011 (3)	0.019 (3)
C14	0.034 (3)	0.060 (5)	0.049 (4)	0.001 (3)	0.008 (3)	0.019 (4)
C15	0.037 (4)	0.053 (4)	0.048 (4)	−0.004 (3)	0.009 (3)	0.020 (3)
C16	0.051 (4)	0.041 (4)	0.059 (5)	0.007 (3)	0.018 (4)	0.025 (4)
C17	0.044 (4)	0.044 (4)	0.057 (5)	0.004 (3)	−0.001 (3)	0.018 (4)
C18	0.087 (6)	0.031 (4)	0.118 (8)	0.006 (4)	−0.004 (6)	0.001 (5)
Mn2	0.0334 (5)	0.0383 (5)	0.0356 (5)	0.0089 (4)	0.0071 (4)	0.0145 (4)
O2	0.058 (3)	0.061 (3)	0.044 (3)	0.028 (2)	0.022 (2)	0.032 (2)
O3	0.085 (3)	0.037 (3)	0.039 (3)	0.015 (2)	0.014 (3)	0.013 (2)
N10	0.040 (3)	0.035 (3)	0.034 (3)	0.005 (2)	0.008 (2)	0.012 (2)
N11	0.057 (4)	0.044 (3)	0.042 (3)	0.004 (3)	−0.005 (3)	0.020 (3)
N12	0.035 (3)	0.062 (4)	0.067 (4)	0.006 (3)	−0.002 (3)	0.018 (3)
N13	0.033 (3)	0.039 (3)	0.044 (3)	0.003 (2)	0.010 (3)	0.009 (3)
N14	0.040 (3)	0.038 (3)	0.034 (3)	0.004 (2)	0.010 (2)	0.012 (2)
N15	0.057 (3)	0.045 (3)	0.053 (4)	0.021 (3)	0.021 (3)	0.026 (3)
N16	0.034 (3)	0.065 (4)	0.037 (3)	0.014 (3)	0.007 (3)	0.013 (3)
N17	0.034 (3)	0.047 (3)	0.035 (3)	0.003 (2)	0.010 (2)	0.020 (3)
C19	0.054 (4)	0.046 (4)	0.045 (4)	0.006 (3)	0.016 (3)	0.023 (3)
C20	0.077 (5)	0.045 (4)	0.039 (4)	0.006 (4)	0.025 (4)	0.017 (3)
C21	0.083 (6)	0.037 (4)	0.037 (4)	0.006 (4)	0.007 (4)	0.014 (3)
C22	0.042 (4)	0.033 (3)	0.036 (4)	0.012 (3)	0.002 (3)	0.006 (3)
C23	0.036 (3)	0.034 (3)	0.038 (4)	0.005 (3)	−0.002 (3)	0.009 (3)
C24	0.031 (4)	0.077 (6)	0.081 (6)	0.003 (4)	0.004 (4)	0.014 (5)
C25	0.050 (4)	0.047 (4)	0.064 (5)	−0.005 (3)	0.026 (4)	0.006 (4)
C26	0.041 (4)	0.049 (4)	0.046 (4)	0.002 (3)	0.018 (3)	0.008 (3)
C27	0.051 (4)	0.042 (4)	0.043 (4)	0.001 (3)	0.013 (3)	0.016 (3)
C28	0.068 (5)	0.041 (4)	0.056 (5)	0.004 (3)	0.028 (4)	0.016 (4)
C29	0.090 (6)	0.041 (4)	0.069 (5)	0.023 (4)	0.045 (5)	0.023 (4)
C30	0.045 (4)	0.039 (4)	0.038 (4)	0.015 (3)	0.017 (3)	0.017 (3)
C31	0.030 (3)	0.049 (4)	0.036 (3)	0.009 (3)	0.007 (3)	0.020 (3)
C32	0.038 (4)	0.072 (5)	0.049 (4)	0.005 (4)	0.013 (3)	0.016 (4)
C33	0.046 (4)	0.059 (5)	0.045 (4)	−0.007 (3)	0.012 (3)	0.009 (4)
C34	0.040 (4)	0.049 (4)	0.036 (4)	0.000 (3)	0.007 (3)	0.021 (3)
Cl1	0.0398 (8)	0.0434 (9)	0.0380 (9)	0.0048 (7)	0.0074 (7)	0.0153 (7)
O4	0.054 (3)	0.077 (4)	0.057 (3)	−0.021 (3)	0.001 (3)	0.030 (3)
O5	0.070 (3)	0.095 (4)	0.087 (4)	0.027 (3)	0.016 (3)	0.069 (4)
O6	0.064 (3)	0.060 (3)	0.080 (4)	0.020 (3)	0.032 (3)	0.023 (3)
O7	0.093 (4)	0.076 (4)	0.041 (3)	−0.013 (3)	0.018 (3)	−0.003 (3)
Cl2	0.0461 (9)	0.0512 (10)	0.0397 (9)	0.0108 (8)	0.0107 (8)	0.0143 (8)
O8	0.066 (3)	0.088 (4)	0.064 (4)	0.025 (3)	0.035 (3)	0.012 (3)
O9	0.084 (4)	0.128 (6)	0.080 (4)	0.008 (4)	−0.006 (3)	0.073 (4)
O10A	0.089 (10)	0.038 (4)	0.068 (5)	−0.001 (4)	0.029 (5)	0.007 (4)
O11A	0.038 (4)	0.092 (10)	0.057 (5)	0.008 (5)	0.021 (4)	0.003 (6)
O10B	0.15 (4)	0.07 (2)	0.11 (2)	0.08 (3)	0.09 (3)	0.065 (18)
O11B	0.13 (3)	0.11 (3)	0.16 (3)	−0.04 (2)	0.02 (2)	0.02 (3)
Cl3	0.0446 (10)	0.0509 (11)	0.0708 (13)	0.0079 (8)	0.0019 (10)	0.0164 (10)
O12	0.081 (5)	0.200 (9)	0.145 (8)	0.023 (5)	−0.040 (5)	0.028 (7)
Cl4	0.0525 (10)	0.0490 (10)	0.0529 (11)	0.0070 (8)	0.0066 (9)	0.0217 (9)
O20	0.051 (3)	0.064 (3)	0.051 (3)	0.001 (2)	−0.004 (2)	0.033 (3)
O21	0.069 (3)	0.081 (4)	0.061 (3)	0.017 (3)	0.021 (3)	0.022 (3)

### Geometric parameters (Å, °)

**Table:**

Mn1—N9	2.170 (6)	N11—C22	1.338 (8)
Mn1—O1	2.182 (4)	N12—C23	1.324 (7)
Mn1—N4	2.236 (4)	N12—C24	1.340 (10)
Mn1—N8	2.254 (5)	N13—C26	1.328 (8)
Mn1—N1	2.259 (5)	N13—C23	1.345 (8)
Mn1—N5	2.271 (5)	N14—C27	1.338 (8)
O1—H1A	0.8400	N14—C30	1.339 (7)
O1—H1B	0.8401	N15—C30	1.320 (7)
N1—C1	1.321 (8)	N15—C29	1.345 (9)
N1—C4	1.338 (7)	N16—C31	1.327 (7)
N2—C4	1.332 (7)	N16—C32	1.346 (9)
N2—C3	1.335 (8)	N17—C31	1.334 (7)
N3—C6	1.331 (8)	N17—C34	1.354 (7)
N3—C5	1.339 (7)	C19—C20	1.359 (9)
N4—C8	1.302 (8)	C19—H19	0.9500
N4—C5	1.351 (7)	C20—C21	1.369 (9)
N5—C12	1.330 (7)	C20—H20	0.9500
N5—C9	1.334 (7)	C21—H21	0.9500
N6—C12	1.330 (7)	C22—C23	1.498 (9)
N6—C11	1.338 (8)	C24—C25	1.355 (11)
N7—C14	1.327 (8)	C24—H24	0.9500
N7—C13	1.328 (7)	C25—C26	1.387 (9)
N8—C13	1.336 (7)	C25—H25	0.9500
N8—C16	1.339 (8)	C26—H26	0.9500
N9—C17	1.115 (8)	C27—C28	1.376 (9)
C1—C2	1.397 (10)	C27—H27	0.9500
C1—H1	0.9500	C28—C29	1.362 (10)
C2—C3	1.366 (10)	C28—H28	0.9500
C2—H2	0.9500	C29—H29	0.9500
C3—H3	0.9500	C30—C31	1.493 (9)
C4—C5	1.478 (8)	C32—C33	1.365 (10)
C6—C7	1.354 (9)	C32—H32	0.9500
C6—H6	0.9500	C33—C34	1.376 (8)
C7—C8	1.397 (9)	C33—H33	0.9500
C7—H7	0.9500	C34—H34	0.9500
C8—H8	0.9500	Cl1—O5	1.412 (5)
C9—C10	1.362 (9)	Cl1—O7	1.422 (5)
C9—H9	0.9500	Cl1—O4	1.431 (5)
C10—C11	1.361 (9)	Cl1—O6	1.438 (5)
C10—H10	0.9500	Cl2—O10B	1.353 (18)
C11—H11	0.9500	Cl2—O8	1.399 (5)
C12—C13	1.488 (8)	Cl2—O11A	1.401 (8)
C14—C15	1.383 (9)	Cl2—O9	1.411 (5)
C14—H14	0.9500	Cl2—O10A	1.476 (8)
C15—C16	1.387 (8)	Cl2—O11B	1.53 (3)
C15—H15	0.9500	Cl3—O12	1.360 (7)
C16—H16	0.9500	Cl3—O15A	1.373 (14)
C17—C18	1.464 (10)	Cl3—O14B	1.383 (13)
C18—H18A	0.9800	Cl3—O13A	1.405 (11)
C18—H18B	0.9800	Cl3—O13B	1.45 (2)
C18—H18C	0.9800	Cl3—O14A	1.479 (12)
Mn2—O3	2.107 (4)	Cl3—O15B	1.58 (3)
Mn2—O2	2.174 (4)	Cl4—O18A	1.356 (15)
Mn2—N13	2.238 (5)	Cl4—O19A	1.37 (2)
Mn2—N10	2.267 (5)	Cl4—O18B	1.40 (2)
Mn2—N14	2.269 (5)	Cl4—O16A	1.419 (12)
Mn2—N17	2.272 (5)	Cl4—O17B	1.420 (13)
O2—H2A	0.8400	Cl4—O19B	1.434 (17)
O2—H2B	0.8400	Cl4—O17A	1.459 (15)
O3—H3A	0.8400	Cl4—O16B	1.466 (13)
O3—H3B	0.8401	O20—H20A	0.8400
N10—C22	1.330 (7)	O20—H20B	0.8400
N10—C19	1.341 (8)	O21—H21A	0.8402
N11—C21	1.311 (9)	O21—H21B	0.8401
			
N9—Mn1—O1	90.14 (19)	C30—N14—Mn2	117.1 (4)
N9—Mn1—N4	95.16 (19)	C30—N15—C29	115.6 (6)
O1—Mn1—N4	91.18 (17)	C31—N16—C32	115.5 (6)
N9—Mn1—N8	92.58 (19)	C31—N17—C34	116.9 (5)
O1—Mn1—N8	89.07 (16)	C31—N17—Mn2	117.1 (4)
N4—Mn1—N8	172.26 (18)	C34—N17—Mn2	125.3 (4)
N9—Mn1—N1	85.1 (2)	N10—C19—C20	122.8 (6)
O1—Mn1—N1	163.05 (16)	N10—C19—H19	118.6
N4—Mn1—N1	73.12 (18)	C20—C19—H19	118.6
N8—Mn1—N1	107.36 (18)	C19—C20—C21	115.3 (7)
N9—Mn1—N5	165.5 (2)	C19—C20—H20	122.3
O1—Mn1—N5	92.60 (17)	C21—C20—H20	122.3
N4—Mn1—N5	98.99 (18)	N11—C21—C20	124.9 (7)
N8—Mn1—N5	73.27 (18)	N11—C21—H21	117.6
N1—Mn1—N5	96.08 (17)	C20—C21—H21	117.6
Mn1—O1—H1A	113.2	N10—C22—N11	125.7 (7)
Mn1—O1—H1B	100.7	N10—C22—C23	116.1 (6)
H1A—O1—H1B	114.1	N11—C22—C23	118.2 (6)
C1—N1—C4	116.4 (6)	N12—C23—N13	126.4 (7)
C1—N1—Mn1	127.0 (4)	N12—C23—C22	117.3 (6)
C4—N1—Mn1	116.5 (4)	N13—C23—C22	116.3 (5)
C4—N2—C3	116.1 (6)	N12—C24—C25	124.5 (7)
C6—N3—C5	116.4 (5)	N12—C24—H24	117.7
C8—N4—C5	116.5 (5)	C25—C24—H24	117.7
C8—N4—Mn1	126.6 (4)	C24—C25—C26	116.1 (7)
C5—N4—Mn1	116.5 (4)	C24—C25—H25	122.0
C12—N5—C9	117.7 (5)	C26—C25—H25	122.0
C12—N5—Mn1	115.6 (4)	N13—C26—C25	121.7 (7)
C9—N5—Mn1	126.6 (4)	N13—C26—H26	119.1
C12—N6—C11	115.1 (5)	C25—C26—H26	119.1
C14—N7—C13	116.3 (5)	N14—C27—C28	121.4 (6)
C13—N8—C16	117.3 (5)	N14—C27—H27	119.3
C13—N8—Mn1	115.7 (4)	C28—C27—H27	119.3
C16—N8—Mn1	125.5 (4)	C29—C28—C27	117.2 (7)
C17—N9—Mn1	169.7 (6)	C29—C28—H28	121.4
N1—C1—C2	121.8 (7)	C27—C28—H28	121.4
N1—C1—H1	119.1	N15—C29—C28	122.8 (6)
C2—C1—H1	119.1	N15—C29—H29	118.6
C3—C2—C1	117.1 (7)	C28—C29—H29	118.6
C3—C2—H2	121.4	N15—C30—N14	126.3 (6)
C1—C2—H2	121.4	N15—C30—C31	117.3 (5)
N2—C3—C2	122.3 (7)	N14—C30—C31	116.4 (5)
N2—C3—H3	118.9	N16—C31—N17	126.2 (6)
C2—C3—H3	118.9	N16—C31—C30	117.7 (6)
N2—C4—N1	126.4 (6)	N17—C31—C30	116.1 (5)
N2—C4—C5	117.2 (5)	N16—C32—C33	123.1 (6)
N1—C4—C5	116.4 (5)	N16—C32—H32	118.4
N3—C5—N4	124.8 (6)	C33—C32—H32	118.4
N3—C5—C4	118.7 (5)	C32—C33—C34	117.3 (7)
N4—C5—C4	116.5 (5)	C32—C33—H33	121.3
N3—C6—C7	123.2 (6)	C34—C33—H33	121.3
N3—C6—H6	118.4	N17—C34—C33	120.9 (6)
C7—C6—H6	118.4	N17—C34—H34	119.6
C6—C7—C8	115.9 (7)	C33—C34—H34	119.6
C6—C7—H7	122.1	O5—Cl1—O7	110.5 (4)
C8—C7—H7	122.1	O5—Cl1—O4	110.4 (3)
N4—C8—C7	123.2 (6)	O7—Cl1—O4	107.9 (3)
N4—C8—H8	118.4	O5—Cl1—O6	108.9 (3)
C7—C8—H8	118.4	O7—Cl1—O6	109.3 (3)
N5—C9—C10	120.7 (6)	O4—Cl1—O6	109.8 (3)
N5—C9—H9	119.6	O10B—Cl2—O8	117.6 (10)
C10—C9—H9	119.6	O10B—Cl2—O11A	62.5 (17)
C11—C10—C9	117.7 (6)	O8—Cl2—O11A	111.9 (4)
C11—C10—H10	121.2	O10B—Cl2—O9	127.2 (13)
C9—C10—H10	121.2	O8—Cl2—O9	112.5 (4)
N6—C11—C10	123.1 (6)	O11A—Cl2—O9	112.5 (5)
N6—C11—H11	118.4	O10B—Cl2—O10A	48.5 (17)
C10—C11—H11	118.4	O8—Cl2—O10A	106.0 (5)
N5—C12—N6	125.6 (6)	O11A—Cl2—O10A	110.6 (6)
N5—C12—C13	117.2 (5)	O9—Cl2—O10A	102.9 (5)
N6—C12—C13	117.2 (5)	O10B—Cl2—O11B	100.7 (18)
N7—C13—N8	125.7 (6)	O8—Cl2—O11B	90.8 (19)
N7—C13—C12	117.7 (5)	O9—Cl2—O11B	94.1 (17)
N8—C13—C12	116.6 (5)	O10A—Cl2—O11B	149.0 (19)
N7—C14—C15	123.1 (6)	O12—Cl3—O15A	119.9 (8)
N7—C14—H14	118.5	O12—Cl3—O14B	109.7 (7)
C15—C14—H14	118.5	O15A—Cl3—O14B	76.6 (8)
C14—C15—C16	116.4 (6)	O12—Cl3—O13A	105.4 (6)
C14—C15—H15	121.8	O15A—Cl3—O13A	112.1 (7)
C16—C15—H15	121.8	O14B—Cl3—O13A	132.3 (7)
N8—C16—C15	121.2 (6)	O12—Cl3—O13B	131.8 (8)
N8—C16—H16	119.4	O15A—Cl3—O13B	67.0 (9)
C15—C16—H16	119.4	O14B—Cl3—O13B	117.8 (9)
N9—C17—C18	179.5 (9)	O13A—Cl3—O13B	45.1 (8)
C17—C18—H18A	109.5	O12—Cl3—O14A	106.8 (6)
C17—C18—H18B	109.5	O15A—Cl3—O14A	111.6 (7)
H18A—C18—H18B	109.5	O13A—Cl3—O14A	98.8 (7)
C17—C18—H18C	109.5	O13B—Cl3—O14A	113.9 (8)
H18A—C18—H18C	109.5	O12—Cl3—O15B	76.7 (10)
H18B—C18—H18C	109.5	O14B—Cl3—O15B	100.7 (11)
O3—Mn2—O2	86.66 (17)	O13A—Cl3—O15B	118.2 (10)
O3—Mn2—N13	101.24 (18)	O13B—Cl3—O15B	86.9 (12)
O2—Mn2—N13	93.91 (19)	O14A—Cl3—O15B	140.8 (10)
O3—Mn2—N10	90.61 (17)	O18A—Cl4—O19A	111.2 (10)
O2—Mn2—N10	165.85 (17)	O18A—Cl4—O18B	56.8 (9)
N13—Mn2—N10	72.99 (19)	O19A—Cl4—O18B	158.0 (12)
O3—Mn2—N14	162.02 (18)	O18A—Cl4—O16A	103.1 (8)
O2—Mn2—N14	85.53 (17)	O19A—Cl4—O16A	113.4 (10)
N13—Mn2—N14	95.43 (18)	O18B—Cl4—O16A	88.2 (10)
N10—Mn2—N14	100.74 (18)	O18A—Cl4—O17B	133.9 (8)
O3—Mn2—N17	93.05 (18)	O19A—Cl4—O17B	69.2 (9)
O2—Mn2—N17	99.04 (17)	O18B—Cl4—O17B	104.9 (10)
N13—Mn2—N17	161.24 (19)	O16A—Cl4—O17B	119.3 (7)
N10—Mn2—N17	94.97 (18)	O18A—Cl4—O19B	60.7 (8)
N14—Mn2—N17	72.26 (18)	O19A—Cl4—O19B	50.7 (9)
Mn2—O2—H2A	126.8	O18B—Cl4—O19B	112.8 (11)
Mn2—O2—H2B	123.2	O16A—Cl4—O19B	128.6 (8)
H2A—O2—H2B	105.2	O17B—Cl4—O19B	100.6 (8)
Mn2—O3—H3A	116.4	O18A—Cl4—O17A	114.3 (9)
Mn2—O3—H3B	124.8	O19A—Cl4—O17A	109.5 (10)
H3A—O3—H3B	118.5	O18B—Cl4—O17A	66.3 (9)
C22—N10—C19	116.1 (6)	O16A—Cl4—O17A	105.2 (8)
C22—N10—Mn2	117.0 (4)	O19B—Cl4—O17A	126.2 (9)
C19—N10—Mn2	126.9 (4)	O18A—Cl4—O16B	118.0 (8)
C21—N11—C22	115.2 (6)	O19A—Cl4—O16B	85.3 (9)
C23—N12—C24	114.6 (7)	O18B—Cl4—O16B	116.3 (10)
C26—N13—C23	116.6 (5)	O17B—Cl4—O16B	108.1 (8)
C26—N13—Mn2	126.0 (5)	O19B—Cl4—O16B	112.5 (9)
C23—N13—Mn2	117.1 (4)	O17A—Cl4—O16B	114.6 (8)
C27—N14—C30	116.6 (5)	H20A—O20—H20B	110.5
C27—N14—Mn2	125.9 (4)	H21A—O21—H21B	85.1
			
N9—Mn1—N1—C1	86.1 (5)	O3—Mn2—N10—C22	97.0 (4)
O1—Mn1—N1—C1	160.2 (6)	O2—Mn2—N10—C22	18.3 (9)
N4—Mn1—N1—C1	−177.0 (6)	N13—Mn2—N10—C22	−4.6 (4)
N8—Mn1—N1—C1	−5.1 (6)	N14—Mn2—N10—C22	−97.1 (4)
N5—Mn1—N1—C1	−79.4 (5)	N17—Mn2—N10—C22	−169.9 (4)
N9—Mn1—N1—C4	−89.4 (4)	O3—Mn2—N10—C19	−82.9 (5)
O1—Mn1—N1—C4	−15.3 (9)	O2—Mn2—N10—C19	−161.6 (6)
N4—Mn1—N1—C4	7.6 (4)	N13—Mn2—N10—C19	175.6 (5)
N8—Mn1—N1—C4	179.5 (4)	N14—Mn2—N10—C19	83.1 (5)
N5—Mn1—N1—C4	105.1 (4)	N17—Mn2—N10—C19	10.3 (5)
N9—Mn1—N4—C8	−97.9 (6)	O3—Mn2—N13—C26	93.6 (5)
O1—Mn1—N4—C8	−7.6 (6)	O2—Mn2—N13—C26	6.3 (5)
N1—Mn1—N4—C8	178.9 (6)	N10—Mn2—N13—C26	−179.2 (5)
N5—Mn1—N4—C8	85.2 (6)	N14—Mn2—N13—C26	−79.6 (5)
N9—Mn1—N4—C5	74.7 (4)	N17—Mn2—N13—C26	−127.4 (6)
O1—Mn1—N4—C5	164.9 (4)	O3—Mn2—N13—C23	−80.6 (4)
N1—Mn1—N4—C5	−8.6 (4)	O2—Mn2—N13—C23	−168.0 (4)
N5—Mn1—N4—C5	−102.3 (4)	N10—Mn2—N13—C23	6.6 (4)
N9—Mn1—N5—C12	19.3 (10)	N14—Mn2—N13—C23	106.2 (4)
O1—Mn1—N5—C12	−81.4 (4)	N17—Mn2—N13—C23	58.3 (8)
N4—Mn1—N5—C12	−173.0 (4)	O3—Mn2—N14—C27	−143.7 (6)
N8—Mn1—N5—C12	6.8 (4)	O2—Mn2—N14—C27	−79.1 (5)
N1—Mn1—N5—C12	113.2 (4)	N13—Mn2—N14—C27	14.4 (5)
N9—Mn1—N5—C9	−163.2 (8)	N10—Mn2—N14—C27	88.1 (5)
O1—Mn1—N5—C9	96.1 (5)	N17—Mn2—N14—C27	179.9 (6)
N4—Mn1—N5—C9	4.5 (5)	O3—Mn2—N14—C30	29.2 (9)
N8—Mn1—N5—C9	−175.6 (6)	O2—Mn2—N14—C30	93.7 (4)
N1—Mn1—N5—C9	−69.3 (5)	N13—Mn2—N14—C30	−172.7 (5)
N9—Mn1—N8—C13	172.0 (4)	N10—Mn2—N14—C30	−99.1 (5)
O1—Mn1—N8—C13	81.9 (4)	N17—Mn2—N14—C30	−7.2 (4)
N1—Mn1—N8—C13	−102.3 (4)	O3—Mn2—N17—C31	−160.4 (4)
N5—Mn1—N8—C13	−11.1 (4)	O2—Mn2—N17—C31	−73.3 (4)
N9—Mn1—N8—C16	6.4 (5)	N13—Mn2—N17—C31	59.8 (8)
O1—Mn1—N8—C16	−83.7 (5)	N10—Mn2—N17—C31	108.8 (4)
N1—Mn1—N8—C16	92.1 (5)	N14—Mn2—N17—C31	9.1 (4)
N5—Mn1—N8—C16	−176.7 (6)	O3—Mn2—N17—C34	9.7 (5)
O1—Mn1—N9—C17	−97 (4)	O2—Mn2—N17—C34	96.8 (5)
N4—Mn1—N9—C17	−6(4)	N13—Mn2—N17—C34	−130.2 (6)
N8—Mn1—N9—C17	174 (4)	N10—Mn2—N17—C34	−81.2 (5)
N1—Mn1—N9—C17	67 (4)	N14—Mn2—N17—C34	179.1 (5)
N5—Mn1—N9—C17	162 (3)	C22—N10—C19—C20	−2.0 (9)
C4—N1—C1—C2	−0.1 (10)	Mn2—N10—C19—C20	177.8 (5)
Mn1—N1—C1—C2	−175.5 (5)	N10—C19—C20—C21	1.9 (10)
N1—C1—C2—C3	0.9 (11)	C22—N11—C21—C20	0.0 (10)
C4—N2—C3—C2	0.5 (10)	C19—C20—C21—N11	−0.8 (10)
C1—C2—C3—N2	−1.1 (12)	C19—N10—C22—N11	1.1 (9)
C3—N2—C4—N1	0.4 (9)	Mn2—N10—C22—N11	−178.7 (5)
C3—N2—C4—C5	−178.5 (6)	C19—N10—C22—C23	−177.8 (5)
C1—N1—C4—N2	−0.6 (9)	Mn2—N10—C22—C23	2.4 (7)
Mn1—N1—C4—N2	175.3 (5)	C21—N11—C22—N10	−0.1 (9)
C1—N1—C4—C5	178.3 (5)	C21—N11—C22—C23	178.8 (5)
Mn1—N1—C4—C5	−5.8 (7)	C24—N12—C23—N13	2.7 (10)
C6—N3—C5—N4	−1.6 (9)	C24—N12—C23—C22	−176.8 (6)
C6—N3—C5—C4	178.7 (5)	C26—N13—C23—N12	−1.9 (9)
C8—N4—C5—N3	2.3 (9)	Mn2—N13—C23—N12	172.9 (5)
Mn1—N4—C5—N3	−171.0 (4)	C26—N13—C23—C22	177.5 (5)
C8—N4—C5—C4	−178.0 (6)	Mn2—N13—C23—C22	−7.7 (7)
Mn1—N4—C5—C4	8.7 (7)	N10—C22—C23—N12	−177.0 (5)
N2—C4—C5—N3	−3.2 (8)	N11—C22—C23—N12	4.0 (8)
N1—C4—C5—N3	177.8 (5)	N10—C22—C23—N13	3.5 (8)
N2—C4—C5—N4	177.1 (5)	N11—C22—C23—N13	−175.5 (5)
N1—C4—C5—N4	−1.9 (8)	C23—N12—C24—C25	−0.5 (11)
C5—N3—C6—C7	−0.7 (10)	N12—C24—C25—C26	−2.1 (12)
N3—C6—C7—C8	1.9 (11)	C23—N13—C26—C25	−1.1 (9)
C5—N4—C8—C7	−0.9 (10)	Mn2—N13—C26—C25	−175.3 (5)
Mn1—N4—C8—C7	171.6 (5)	C24—C25—C26—N13	2.9 (10)
C6—C7—C8—N4	−1.1 (11)	C30—N14—C27—C28	0.3 (9)
C12—N5—C9—C10	−0.5 (9)	Mn2—N14—C27—C28	173.2 (5)
Mn1—N5—C9—C10	−178.0 (5)	N14—C27—C28—C29	0.7 (11)
N5—C9—C10—C11	0.3 (10)	C30—N15—C29—C28	2.4 (11)
C12—N6—C11—C10	−1.1 (10)	C27—C28—C29—N15	−2.1 (11)
C9—C10—C11—N6	0.6 (11)	C29—N15—C30—N14	−1.3 (10)
C9—N5—C12—N6	−0.1 (9)	C29—N15—C30—C31	−179.7 (6)
Mn1—N5—C12—N6	177.7 (5)	C27—N14—C30—N15	0.0 (10)
C9—N5—C12—C13	180.0 (5)	Mn2—N14—C30—N15	−173.5 (5)
Mn1—N5—C12—C13	−2.3 (7)	C27—N14—C30—C31	178.5 (5)
C11—N6—C12—N5	0.8 (9)	Mn2—N14—C30—C31	4.9 (7)
C11—N6—C12—C13	−179.2 (6)	C32—N16—C31—N17	0.9 (10)
C14—N7—C13—N8	−0.3 (9)	C32—N16—C31—C30	−179.8 (6)
C14—N7—C13—C12	−179.4 (6)	C34—N17—C31—N16	−1.3 (9)
C16—N8—C13—N7	1.6 (9)	Mn2—N17—C31—N16	169.6 (5)
Mn1—N8—C13—N7	−165.2 (5)	C34—N17—C31—C30	179.4 (5)
C16—N8—C13—C12	−179.4 (5)	Mn2—N17—C31—C30	−9.7 (7)
Mn1—N8—C13—C12	13.8 (7)	N15—C30—C31—N16	2.4 (9)
N5—C12—C13—N7	171.4 (5)	N14—C30—C31—N16	−176.2 (6)
N6—C12—C13—N7	−8.6 (8)	N15—C30—C31—N17	−178.2 (6)
N5—C12—C13—N8	−7.7 (8)	N14—C30—C31—N17	3.2 (8)
N6—C12—C13—N8	172.3 (5)	C31—N16—C32—C33	1.4 (10)
C13—N7—C14—C15	−2.3 (10)	N16—C32—C33—C34	−3.1 (11)
N7—C14—C15—C16	3.5 (10)	C31—N17—C34—C33	−0.6 (9)
C13—N8—C16—C15	−0.2 (9)	Mn2—N17—C34—C33	−170.6 (5)
Mn1—N8—C16—C15	165.2 (5)	C32—C33—C34—N17	2.6 (10)
C14—C15—C16—N8	−2.1 (10)		

### Hydrogen-bond geometry (Å, °)

**Table:**

*D*—H···*A*	*D*—H	H···*A*	*D*···*A*	*D*—H···*A*
O1—H1A···N6^i^	0.84	2.49	3.139 (6)	135
O1—H1A···N7^i^	0.84	2.15	2.923 (6)	152
O1—H1B···O21^i^	0.84	1.89	2.695 (7)	160
O2—H2A···N15^ii^	0.84	2.59	3.244 (7)	136
O2—H2A···N16^ii^	0.84	2.25	3.012 (7)	151
O2—H2B···O11A^iii^	0.84	2.01	2.809 (10)	158
O3—H3A···O20	0.84	1.79	2.614 (6)	166
O3—H3B···O4^iii^	0.84	2.07	2.861 (6)	157
O20—H20A···N2^iv^	0.84	2.08	2.903 (7)	168
O20—H20A···N3^iv^	0.84	2.56	3.066 (7)	120
O20—H20B···O6	0.84	2.08	2.871 (7)	157
O21—H21A···O12^v^	0.84	2.43	3.126 (12)	141
O21—H21B···O16A^iv^	0.84	2.07	2.904 (13)	172

Symmetry codes: (i) −*x*+1, −*y*+1, −*z*+1; (ii) −*x*+1, −*y*+2, −*z*; (iii) −*x*+1, −*y*+1, −*z*; (iv) *x*+1, *y*, *z*; (v) −*x*+1, −*y*, −*z*+1.
